# Association between birth season and physical development in children under 3 years old residing in low-income counties in western China

**DOI:** 10.1371/journal.pone.0187029

**Published:** 2017-11-14

**Authors:** Fangliang Lei, Shanshan Li, Baibing Mi, Danmeng Liu, Jiaomei Yang, Pengfei Qu, Ruo Zhang, Xiaofeng Zhang, Jia Ying, Shaonong Dang, Hong Yan

**Affiliations:** 1 Department of Epidemiology and Health Statistics, School of Public Health, Xi’an Jiaotong University Health Science Center, Xi’an, Shaanxi, China; 2 Assisted Reproduction Center, Northwest women and children’s Hospital, Xi’an, Shaanxi, China; 3 Luoyang Maternity and Child Care Hospital, Luolong District, Luoyang, Henan, China; 4 Department of Disease Prevention and Control, Putuo Center for Disease Prevention and Control, Zhoushan, Zhejiang, China; 5 Xi’an Jiaotong University, Health Science Center, Xi’an, Shaanxi, China; 6 Nutrition and Food Safety Engineering Research Center of Shaanxi Province, Xi’an, Shaanxi, China; 7 Key Laboratory of Environment and Genes Related to Diseases (Xi’an Jiaotong University), Ministry of Education, Xi’an, Shaanxi, China; University of Michigan, UNITED STATES

## Abstract

**Objective:**

To explore the association between birth season and physical development and provide a necessary reference value to inform the implementation of public health services.

**Design:**

Cross-sectional study.

**Setting:**

Forty-five counties in ten provinces in western China in 2005.

**Subjects:**

A sample of 13,387 children under 3 years old and their mothers were recruited using a stratified, multistage, cluster random sampling method.

**Results:**

The results of the circular distribution analysis suggested that stunting and underweight exhibited time aggregation (Z = 32.57, P<0.05; Z = 10.42, P<0.05) among children under 3 years old. The *Z* − value for wasting, however, was not statistically significant (P>0.05). The generalized linear mixed models showed that children born in the summer were less likely to exhibit stunting (OR: 0.74~0.97) than were children born in the winter after adjusting for confounders, but no significant differences were identified for the other seasons. In addition, among children aged 25 to 36 months, those born in the summer and autumn were less likely to exhibit stunting after adjusting for confounders than were children born in the winter, but the association between birth in spring and stunting was not statistically significant.

**Conclusions:**

Stunting was associated with season of birth among children under 3 years old in low-income counties in western China, especially children aged 25 to 36 months, and children born in the summer and autumn were less likely to exhibit stunting than were children born in the winter.

## Introduction

Seasonal patterns have been identified in childhood growth parameters, including birth weight and length[1–5] and body size in both children[6] and adults[7, 8], and both developing and developed countries. However, some variation exists in the seasonal patterns of growth in different countries. For example, an October—April peak and May—September nadir were reported for height among children in north Poland[5, 6], while in Dunedin, New Zealand, birth weight and length were found to be highest among those with October (spring) births and lowest among those with January (summer) births[8]. Furthermore, dual peaks and nadirs have been identified in Japan[9, 10] and southern Chile[4]; however, this may reflect, in part, concomitant seasonal variation in gestation length[9, 10]. In addition, Emma Pomeroy’s research[11] demonstrated associations between birth month and stature among rural highland Peruvian children aged 6 months to 8 years, with a peak observed in spring births(October-November). D. Schwekendiek reported that cohorts born in autumn were taller in North Korea[12]. A Chinese study suggested that the seasonal effects on weight gain and length gain were largely independent in Shanghai; in addition, infants tended to grow faster in height in spring and summer and faster in weight and BMI in autumn and winter[13]. The different conclusions of the aforementioned studies might have resulted from various factors, including regional and study population differences.

However, little research has been conducted regarding the relationship between birth season and physical development among children in rural counties in western China. In addition, the majority of previous studies have utilized correlation or regression analyses, while our research adopted a combination of the circular distribution method and generalized linear mixed models approach to innovatively analyze this association. The circular distribution method[14–16]may be used to convert periodic change data into linear data using a trigonometric function to facilitate the performance of a simple statistical analysis. Next, the generalized linear mixed model approach was applied to analyze data that demonstrated a hierarchical structure in the sampling investigations. Therefore, the aim of this study was to explore the relationship between birth season and physical development in rural counties in western China using a novel statistical approach and provide a necessary reference value to inform the implementation of public health services.

## Method

### Participants and design

The present cross-sectional study was conducted as part of the Rural Primary Health Care Project, which was conducted in 2005 in rural western China and included a total of 14,112 women and children from 45 counties in ten provinces. Considering the hierarchical structure of Chinese administrative districts and the imbalanced population distributions among the different provinces, a stratified, three-stage, probability-proportional-to-size sampling method was employed in the present study. The included counties were determined by the Chinese Ministry of Health and UNICEF. Forty-five counties were selected in terms of social and economic development by MOH and UNICEF. All counties were poor economy and higher willing-ness to participate in the project. Five townships were randomly selected in each county; four villages were randomly selected in each township, and then a woman with children who were younger than 3 years old was selected from sixteen randomly selected families in each village. All women were interviewed in person by trained professional interviewers from the Xi’an Jiaotong University College of Medicine. Women who agreed to participate in the study signed the consent form. Relatives or neighbours signed on behalf of illiterate participants after their verbal consent. The ethics committee of the Xi’an Jiaotong University College of Medicine approved the study.

Seven hundred and twenty-five observations were removed because of the presence of unreasonable data. These observations had either Z-scores of height(length) for age, weight for age and weight for height that were not with in the normal cut-off values[17, 18] or were for children who were older than 36 months. Thus, a total of 13,387 observations were analyzed (S1 File).

### Measures

#### Physical development evaluation index

The recumbent lengths of all infants and children in the study were measured with a standard calibrated board accurate to the nearest 1mm (Model WB-II, Beijing Tractor Company No. 6 Measuring Factory, Beijing, China). Tared weighting was used to measure child’s weight. Mother was to be weighted first alone, and mother with handing child was to be weight secondly. The difference of twice weight was child’s weight. All children were weighted with minimal clothing. All weights were measured to the nearest 500 g with a standard calibrated balance scale (Model YGZ-12, Wuxi Measurement Factory, Wuxi, China). Measurements were performed according to recommended standard methods by trained interviewers.

Z-scores were used to evaluate the physical status of included infants. Based on the 2006 WHO criteria for sex- and age-specific height and weight, Z-scores (HAZ, WAZ, WHZ) for height(length) for age, weight for age and weight for height were calculated. The formula used to calculate these z values was as follows: Z = (physical measurement-median of the WHO standard for children of the same age and sex)/standard deviation of the WHO standard. The presence of a Z value for height for age greater than 2 standard deviations below the mean was defined as stunting. The presence of a Z value for weight for age greater than 2 standard deviations below the mean was defined as underweight. The presence of a Z value for weight for height greater than 2 standard deviation below the mean was defined as wasting.

#### Control variables

Controlling for potential confounding factors was necessary when determining the relationship between birth season and physical development. Based on the currently available body of knowledge and the nature of our data, we selected potential confounding factors from four groups of variables: children, family, fathers and mothers. The factors included within the children group were the child’s sex and age. The factors included within the family group were the total number of family members and total number of children. The factors included within the mother group included maternal education level, age at the child’s birth, height and weight. The factors included within the father group included paternal education level. Finally, whether the children were breastfed and whether the mother looks after the child were also included. We selected the control variables based on the following considerations:(i)whether the aforementioned factors affected the physical development of the children (factors that were primarily based on related studies[19, 20]); and (ii) how these variables differed between participants across seasons, which was determined during the data analysis.

#### Quality control

The interviewers were trained before administering the survey. A pilot survey was performed before the formal survey was administered to acquire necessary information and to test the formal survey. During the survey, a checking system was utilized, wherein the investigators checked the data while in the field, and another interviewer and the supervisor also checked the data. The participants were re-interviewed when either logistical questions or missing values were found. The accurate age of each child was collected from the Permanent Residence Registration and/or the Record of Planned Immunization where the birth data were recorded.

#### Statistical analysis

A database was designed using EpiData version 3.02, and data entry was duplicated. Initially, the descriptive statistics for the characteristics of participants were summarized using means±SDs and medians with interquartile ranges (IQRs) for normally and abnormally distributed continuous variables, respectively. Count and proportions were used for describing categorical variables. The *χ*^*2*^ test was used to analyze differences in proportions between groups. The ANOVA test was used to analyze differences in continuous variables between groups.

The circular distribution method is generally used to analyze periodic things, such as infectious diseases. It is not clear whether there are seasonal differences in the physical development of infants and young children. So the circular distribution method was used to analyze the seasonal distribution of poor physical development among children under 3 years old. In statistics, a circular distribution serves as the probability distribution of a random variable whose values are angles, usually taken to be with in the following range: [0, 2*π*). The circular distribution method may be used to convert periodic change data into linear data using a trigonometric function[14–16]. When using this method, the 12 months are seen as a circumference(360 degrees). We obtained monthly median values to complete the calculation of the circular distribution. The central tendency was expressed by an average angle. The average angle calculated using a relevant formula that was converted into a corresponding date. The date was the highest incidence of poor physical development among children under 3 years old. The following formula was used: *X* = (∑*f*_*i*_ *cos α*_*i*_)/*n*, *Y* = (∑*f*_*i*_ *sin α*_*i*_)/*n*, $r\;=\;\sqrt{X^{2}+Y^{2}},\;\sin\;\overset{-}{\alpha }\;=\;Y/r,\;\cos\;\overset{-}{\alpha }\;=\;X/r$. The degree of dispersion of the circular distribution data was expressed using the sample statistic *r*, which was also regarded as the discrete level indicator of the angle. The Rayleigh’s test was used to examine each average angles for the sample. A critical value for the Rayleigh’s test (Z) was used to judge whether each average angle was statistical significant, through the calculation of the following test statistic: *Z* = *nr*^2^.

Because of the multilevel hierarchical structure of the data[21], the generalized linear mixed model approach was used, which is a good method for analyzing data with a hierarchical structure and can be applied in sampling investigations. Finally, a 2-level analysis was performed to adjust for the effect of randomization by counties and to analyze the associations between birth season and the different physical development parameters with county to level 2 and individual to level 1 by ten potential confounding factors. The regression coefficients and their 95% confidence intervals (95%CI) were computed for the spring, summer, and autumn using winter as a reference. Four adjusted models containing these covariates were constructed to systematically control for these cofounders. Model 1 adjusted for the age and sex of infants and young children. Model 2 adjusted for the variables in model 1 plus the relevant maternal and paternal characteristics, including the mother’s height, weight, age at the child’s birth, and education level and the father’s education level. Model 3 adjusted for the variables in model 2 and family demographic characteristics (including both the total numbers of family members and children). Model 4 adjusted for the variables in model 3 plus whether the mother looks after the child.

All statistical analyses were performed using SAS 9.3(SAS Institute Inc., Cary, NC) and Excel 2007. A two-tailed P<0.05 was considered statistically significant.

## Result

### Sociodemographic characteristics of participants

The details of the sample and distribution of the major demographic variables are shown in Table 1. A total of 14,112 women and children were enrolled in the study. After excluding the 725 observations with unreasonable information, 13,387 children (57.43% male) and mothers were included in the present analysis. In total, 3030, 2840, 3821 and 3696 of the included children were born in the spring, summer, autumn and winter, respectively. In addition, the number of children and constituent ratios are shown in Table 2 by age group and season.

**Table 1:** Sociodemographic characteristics in rural western China, 2005.

Characteristics		Season of birth				P
		Spring (Mar—May)	Summer (Jun—Aug)	Autumn (Sep—Nov)	Winter (Dec—Feb)	
Number of participants, n		3030	2840	3821	3696	
Age of infants and young children (months)	mean±SD	14.86±9.46	19.63±9.85	18.89±9.79	16.65±9.55	<0.001
Sex of infants and young children, n(%)						
Boy		1706(56.30%)	1686(59.37%)	2173(56.87%)	2123(57.44%)	0.093
Girl		1324(43.70%)	1154(40.63%)	1648(43.13%)	1573(42.56%)	
Maternal age at the child’s birth (years)	mean±SD	25.37±4.71	25.38±4.78	25.18±4.64	25.26±4.65	0.224
Mother's height(cm)	mean±SD	155.19±5.32	155.23±5.22	155.36±5.08	155.20±5.26	0.467
Mother's weight(kg)	mean±SD	52.58±7.56	51.82±6.88	52.12±7.02	52.15±7.16	0.001
Mother's education(years)	mean±SD	6.48±3.13	6.37±3.13	6.52±3.15	6.48±3.19	0.302
Father's education(years)	mean±SD	7.55±2.65	7.58±2.66	7.67±2.65	7.62±2.71	0.318
Family population	M(Q1,Q3)	5(4,6)	5(4,6)	5(4,6)	5(4,6)	
Children population	M(Q1,Q3)	1(1,2)	1(1,2)	1(1,2)	1(1,2)	
Children population by mothers looked after, n(%)		2545(80.99%)	2145(75.53%)	2903(75.97%)	2924(79.11%)	<0.001
Children population of breastfeeding, n(%)		2895(95.54%)	2709(95.39%)	3671(96.07%)	3527(95.43%)	0.454

**Table 2:** The number of children for every season among different age group.

Month	Season of birth(%)			
	Spring	Summer	Autumn	Winter
0~12	979(18.62)	1208(22.97)	1665(31.67)	1406(26.74)
13~24	1192(24.96)	992(20.77)	1224(25.63)	1368(28.64)
25~36	859(25.62)	640(19.08)	932(27.80)	922(27.50)

### Peak time of poor physical development

**Fig 1:**
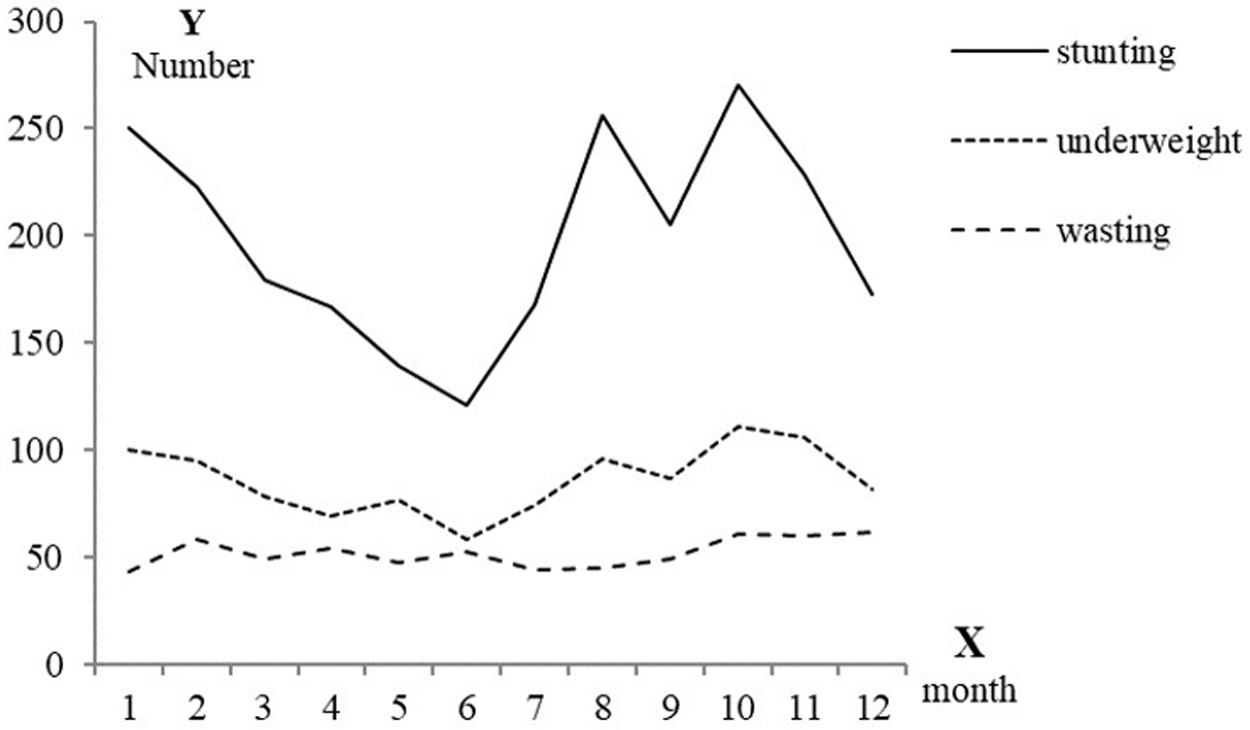
The month distribution of poor physical development.

The details of the distribution of poor physical development are shown in Fig 1. From Fig 1, we can conclude that the occurrence of stunting and underweight exhibited times of peak values, while wasting occurred with equivalent frequency during each month. The numbers of stunting and underweight children born in the autumn and winter were far greater than those born during the other seasons, and the numbers of children born in the summer (in June) who exhibited stunting or were underweight were the fewest. Moreover, we used the circular distribution to describe and statistically test these distributions. Using this method, the 12 months were viewed as a circumference(360 degrees) of the circular distribution. Each month comprised 30 degrees. We obtained monthly median value to completed the calculations for the circular distribution method. Relevant values for the evaluated poor physical development indices are shown in Table 3. The occurrence of stunting and underweight were temporally aggregated (Z = 32.57, P<0.05; Z = 10.42, P<0.05) among children under 3 years old in rural western China in 2005. The *Z* − value for wasting, however, was not statistically significant(P>0.05). The average angles were converted into corresponding dates. The peak time of stunting was in mid-November, and 95% confidence interval for this peak time was from the beginning to the middle of November. The peak time of underweight was mid-November, and 95% confidence interval for this peak time was the whole month of November.

**Table 3:** The monthly occurrence status of poor physical development index.

Poor physical development	$\bar{\alpha }\pm s$	Z	P	95%CI	*r*
Stunting	312.94±118.69	32.57	<0.05	305.65~320.23	0.12
Underweight	317.61±122.83	10.42	<0.05	306.16~329.06	0.10
Wasting	341.60±140.82	1.49	>0.05	-	-

### Association between birth season and physical development

In Table 4, the GLMM results obtained for physical development as an outcome variable represent the association between birth season and physical development. The group of children born in the winter group were used as a reference group, and the children born during other seasons were compared with the winter births. After adjusting for all variables from model 1 to model 4, the *β*-estimates showed that physical development was associated with birth season. Furthermore, the four *β*-estimates were similar, which indicated that the models were stable. Specifically, a statistically significant association between birth season and the stunting was found. Children born in the summer are less likely to exhibit stunting(OR: 0.74~0.97) than were those born in the winter, but differences were identified for children born in the other seasons. However, the associations between birth season and the other physical development indices demonstrated no significant differences(P>0.05).

**Table 4:** Association between birth season and physical development of children under 3 years old using GLMM.

Physical development	Birth season	Model 1		Model 2		Model 3		Model 4	
		*β*(95%CI)	P	*β*(95%CI)	P	*β*(95%CI)	P	*β*(95%CI)	P
Stunting	Spring	-0.03(-0.16~0.11)	0.72	-0.02(-0.16~0.12)	0.77	-0.03(-0.17~0.11)	0.69	-0.03(-0.17~0.11)	0.68
	Summer	**-0.16(-0.30~-0.02)**	**0.02**	**-0.16(-0.30~-0.02)**	**0.03**	**-0.17(-0.31~-0.03)**	**0.02**	**-0.17(-0.31~-0.03)**	**0.02**
	Autumn	-0.10(-0.23~0.03)	0.12	-0.10(-0.23~0.03)	0.13	-0.10(-0.23~0.03)	0.14	-0.10(-0.23~0.03)	0.14
	Winter	0		0		0		0	
Underweight	Spring	0.04(-0.15~0.23)	0.70	0.04(-0.15~0.23)	0.68	0.04(-0.16~0.23)	0.71	0.04(-0.16~0.23)	0.71
	Summer	-0.07(-0.27~0.12)	0.44	-0.07(-0.26~0.13)	0.50	-0.07(-0.26~0.12)	0.47	-0.07(-0.26~0.12)	0.47
	Autumn	0.02(-0.16~0.20)	0.84	0.02(-0.16~0.20)	0.83	0.02(-0.16~0.20)	0.83	0.02(-0.16~0.20)	0.81
	Winter	0		0		0		0	
Wasting	Spring	0.09(-0.14~0.33)	0.43	0.11(-0.13~0.34)	0.37	0.11(-0.13~0.34)	0.38	0.11(-0.13~0.34)	0.37
	Summer	0.16(-0.08~0.40)	0.18	0.16(-0.08~0.40)	0.18	0.16(-0.08~0.40)	0.19	0.16(-0.08~0.40)	0.19
	Autumn	0.08(-0.15~0.31)	0.49	0.08(-0.15~0.30)	0.51	0.08(-0.15~0.30)	0.51	0.08(-0.15~0.30)	0.51
	Winter	0		0		0		0	

CI = confidence interval; GLMM = Generalized linear mixed models; values are *β*-estimates (95% CI).

Coefficients significant at the 5% level are bold and underlined. The 95% confidence intervals are obtained using 1000 bootstrap replications.

#Model adjustments: model1: adjusted for age and sex of infants and young children. model 2: adjusted for the variables in model 1 plus mother's relevant characteristics and father's education level, including mother's height, weight, maternal age at the child’s birth, mother and father's education level. Model 3: adjusted for the variables in model 2 and for family demographic characteristics (including both family population and children population). Model 4: adjusted for the variables in model 3 plus whether the mother looks after child. All these models use the children who were born in winter as reference.

### Association between birth season and physical development for different age groups

**Fig 2:**
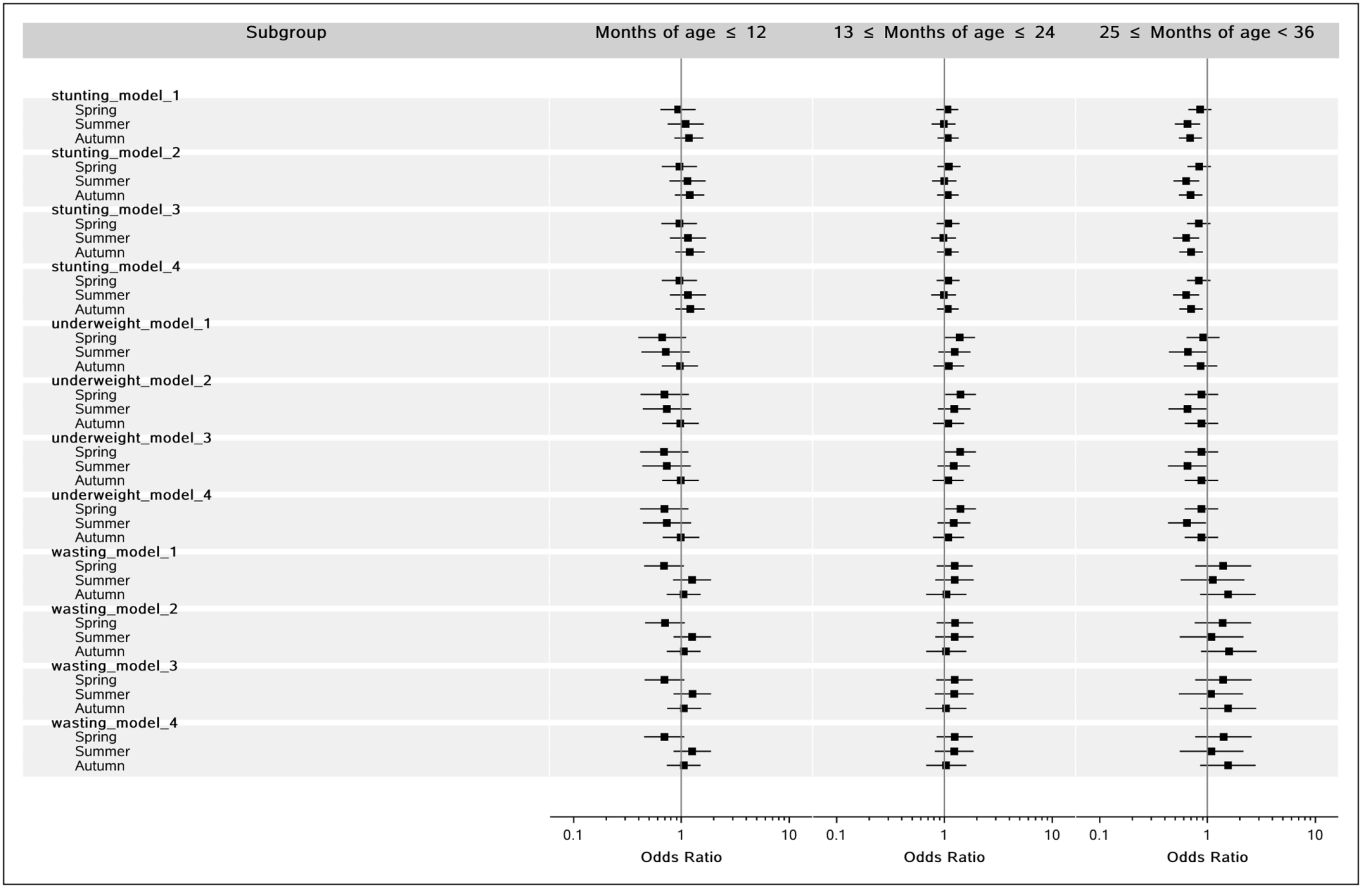
This figure illustrates the odds ratio and 95% confidence intervals of the association between birth season and physical development for different age group.

Fig 2 shows the association between birth season and physical development for different age groups. In each age group, the results of the 4 models corresponding to each outcome measure were similar, which indicated the stability of these models. Specifically, for children aged 25 to 36 months, children born in the summer and autumn are less likely stunting, compared to the children born in the winter, but the relationship between spring and stunting had not found significant difference. In addition, for other age groups, the associations between birth season and the other physical development indices were not significantly different(P>0.05).

## Discussion

### The main methods and conclusion

In this study, the circular distribution method was used to analyze and compare periodic data. The chi-square test is not considered an appropriate method of analysis for periodic data, because it is usually used to test whether there are differences between two or more rates (or constituent ratios), or whether two variables are correlated [22]. Using the circular distribution, we concluded that stunting and underweight was associated with season of birth, while wasting was independent of the effect of season. The peak times of stunting and underweight were similar, with both development indices peaking in mid-November (late Autumn). However, the circular distribution method only provides a simple statistical description that may be used to assess the association between birth season with physical development but does not allow for adjustment for other variables. Therefore, the use of the GLMM method overcame this limitation. The GLMM models were systematically generated to control for potential cofounders (including the child’s sex and age; numbers of family members and children; maternal and paternal education level; maternal age at the child’s birth, height and weight; whether the child was breastfed; and whether the mother looks after child) and, therefore, explain the independent associations between the physical development indices and the season of birth.

In our study, we found that the stunting was associated with birth season among the children aged 25 to 36 months in low-income counties in western China and that children born in the summer and autumn were less likely exhibit stunting than were those born in the winter. However, regardless of the age group assessed, the associations between birth season and other physical development indices were nor significantly different. Four adjusted models containing the potentially confounding covariates were systematically established. In addition, the results obtained from the different models were approximately the same, which indicated the stability of the models.

### Comparisons with other studies and implications of findings

The effect of birth season on physical development among infants and young children has been previously investigated in a few studies conducted elsewhere[12, 23, 24]. D. Schwekendiek[12] analyzed data from 1,978 preschool boys exposed to North Korea’s food crisis in the 1990s and found that those born in fall (September—November)were tallest, while those born in spring (March—May)were shortest. A conducted in China showed that birth month had some association with attained size in Shanghai[13]. F. Gloria-Bottini's research suggested that the environmental and genetic factors that favor reproduction in the early months of the year also favor the birth of well-nourished offspring in the Caucasian population in Central Italy[24]. In addition, while there was a clear seasonal pattern in birth weight in Denmark during 1936–1989, this pattern changed across the study period[25]. The aforementioned studies illuminated the relationship between physical development and birth season and provided some references and evidence for our research.

The present cross-sectional study indicated the presence of an association between birth season and stunting in children aged 25 to 36 months; however, the causality behind this association is not clear. A previous study reported that the effects of warmer environmental temperatures during gestation could prevent fluctuations in maternal nutrition and, hence, stimulate fetal growth[26]. Indirectly, seasonal fluctuations can impact health status via nutrition (e.g., availability of seasonal food products such as fruit), energy expenditure (e.g., work load may vary across seasons in agrarian societies), and disease exposure (e.g., respiratory viruses may be more prevalent in winter and vectors for malaria have seasonal breeding cycles)[27]. These reports are in agreement with the conclusions of the current study. In the present study, after adjusting for relevant socio-demographic and anthropometric parameters, the variation in the β-estimates for the associations between birth season and the evaluated physical development indices were stable in models 1 to 4. This result may indicate that these factors are not the primary explanations for the effect of physical development.

### Strengths and limitations

In present study, weight, height and age were transformed into Z-scores (WHZ, WAZ, HAZ) for height(length) for age, weight for age and weight for height according to the 2006 WHO sex-and age-specific standards for height and weight. Thus, these Z-scores (WHZ, WAZ, HAZ) were representative of stunting, underweight and wasting, which were defined according to their relevant cut-off points. In detail, stunting, underweight and wasting were considered to comprehensively evaluate the physical development of children. Furthermore, other all relevant studies have adopted correlation or regression analyses to examine the association between physical development and birth season. However, the current study innovatively and rationally used the circular distribution method to illuminate relationships in periodic data. Moreover, the GLMM models adjusted for relevant covariates were generated to further elucidate the association between stunting and birth season. No association between birth season and underweight or wasting was identified.

This study also had some limitations. First, some major confounders, including physical activity, social stimulation[28, 29], family income, and parental occupation, were not adjusted for because we lacked these data. In addition, the reproductive cycles in plants and animals are synchronized with seasonal changes, and variations in the intensity of sun exposure represented a crucial factor in the development of such cycles. The intensity of sun exposure progressively increases during the first month of year, reaching a plateau in middle months and decreasing progressively during the last months of the year[30]. Similarly, this finding is compatible with a hypothesis that seasonality affects physical development through different mechanisms. Therefore, a limitation of this study was that the mechanism through which seasonality affects physical development needs to be further clarified in the future.

## Supporting information

S1 File Data information. (ZIP)
