# Supplementary material for: Association between birth season and physical development in children under 3 years old residing in low-income counties in western China
Source: PLoS One. 2017 Nov 14;12(11):e0187029. doi: 10.1371/journal.pone.0187029 (PMC5685582; doi:10.1371/journal.pone.0187029)
Supplement: S1 File — (ZIP) [file pone.0187029.s001.zip › supporting Information/Variable assignment table.docx]

| Variables | [Assignment](javascript:void(0);) | |
| --- | --- | --- |
| Stunting | 0=No | 1=Yes |
| Underweight | 0=No | 1=Yes |
| Wasting | 0=No | 1=Yes |
| Sex of children | 0=Girl | 1=Boy |
| Child by mother looked after | 0=Yes | 1=No |
| Child of breastfeeding | 0=No | 1=Yes |
| Nationality | 0=Han | 1= Minority |
| Season of birth | 1=Spring | 2=Summer |
|  | 3=Fall | 4=Winter |

Variable assignment table
